# Induction of spontaneous curvature and endocytosis: Unwanted consequences of cholesterol extraction using methyl-β-Cyclodextrin

**DOI:** 10.1080/19420889.2018.1444306

**Published:** 2018-03-13

**Authors:** Takashi Hirama, Gregory D. Fairn

**Affiliations:** a Division of Respirology, Department of Medicine, University of Toronto, Toronto, ON, Canada; b Program of Lung Transplant, Multi-Organ Transplant Program, Toronto General Hospital, Toronto, ON, Canada; c Nontuberculous Mycobacteria Program, Division of Respirology, Toronto Western Hospital, Toronto, ON, Canada; d Keenan Research Centre for Biomedical Science, St. Michael's Hospital, Toronto, ON, Canada; e Department of Biochemistry, University of Toronto, Toronto, ON, Canada; f Department of Surgery, University of Toronto, Toronto, ON, Canada

**Keywords:** Cholesterol, Cyclodextrin, Phosphatidylserine, Plasma membrane, Spontaneous Curvature

## Abstract

Membrane curvature is a property of biological membranes essential for organelle morphology and the formation of tubulovesicular carriers. Curvature generation is influenced by the lipid composition of the membrane and protein-mediated processes. Lipids with small headgroups, such as phosphatidic acid, are conical and impose negative curvature on a monolayer. Conversely, lipids with large headgroups relative to the hydrophobic tail(s), such as lysophosphatidylcholine, have an inverted conical shape and impose positive curvature. Due to its abundance and high rates of spontaneous flip-flop between membrane leaflets cholesterol is proposed to buffer the formation of membrane curvature. Recently, we demonstrated that cholesterol is also crucial for maintaining the proper spacing of anionic phospholipids. Upon extraction of cholesterol with cyclodextrin there is a sharp increase in the negative surface charge density of the plasma membrane, which promotes electrostatic repulsion between anionic headgroups, the generation of spontaneous positive curvature and rapid membrane internalization.

The plasma membrane (PM) is comprised of a lipid bilayer that serves as not only a barrier but also a site for cellular transactions including endocytosis, exocytosis, and signal transduction. An intriguing feature of the PM is the asymmetric distribution of lipids between the cytosolic and exofacial leaflets [1]. Anionic glycerophospholipids, including phosphatidic acid, phosphatidylinositol and its phosphorylated derivatives, and phosphatidylserine (PtdSer) are primarily restricted to the cytosolic leaflet of the PM in resting cells. Thus, the inner leaflet of the PM possesses a significant and biologically relevant negative surface charge [2]. Conversely, the lipids in the exofacial leaflet are mainly uncharged or zwitterionic and thus the surface of this leaflet of the bilayer is close to zero. In addition to phospholipids, sterols, predominantly cholesterol in mammalian cells, are essential components of the PM and required to maintain its biophysical properties such as fluidity, thickness, and spontaneous curvature [3].

Curiously, PtdSer and cholesterol display a mirrored distribution within cells; both are synthesized in the endoplasmic reticulum (ER), yet are enriched in the PM [4]. While at the nanoscale level Chol-PtdSer rich domains have been described to influence K-Ras signaling [5]. Additionally, both lipids are enriched in and required for caveola formation or stability [6,7]. We recently reported that PtdSer containing of stearate and oleate as its acyl chains interacts with Chol in giant unilamellar vesicles and cholesterol oxidase protection assays [8]. While at the cellular level PtdSer is required to retain cholesterol in the cytosolic leaflet of PM [8]. Despite the growing number of studies indicating a requirement for PtdSer to maintain Chol in the inner leaflet of the PM the relationship between PtdSer and Chol is not well understood. Importantly, it has been unclear if Chol impacts the distribution of PtdSer.

On a per mole basis, Chol and glycerophospholipids are estimated to be roughly equivalent and together comprise about 80 mol% of the plasmalemmal lipids [9]. Therefore, the rapid extraction of Chol using high concentrations of methyl-b-cyclodextrin allowed us to examine the role of Chol in maintaining the distribution of PtdSer. Using a genetically encoded biosensor for PtdSer as well as PM isolation and lipid determination we found that within minutes of extracting the plasmalemmal Chol a significant fraction of the PtdSer was internalized to endosomal compartments [10]. As Chol constitutes a significant fraction of the PM lipids, we suspected that its removal would cause increased lateral packing of PtdSer and other lipids in the PM. In a resting cell, ≈20% of the cytosolic leaflet lipids possess negative charge and this has been estimated to be increased to ≈30% following Chol extraction see (Fig. 1) [10]. Based on the notion, we proposed that the close packing of negatively charged headgroups would have a consequence of increasing electrostatic repulsion of the negatively charged headgroups in the inner leaflet and promote spontaneous membrane inward curvature (Fig. 1a) [10]. Indeed, theoretical calculations support the notion that even subtle increases in negative charge density could have a substantial impact on the degree of spontaneous curvature (Fig. 1b, c) [10]. At the cellular level, the generation of spontaneous membrane curvature can be recognized and stabilized by BAR domain proteins such as endophilin.

To verify the generation of spontaneous curvature as a consequence of electrostatic repulsion, we presented four lines of evidience [10]. First, red blood cell ghosts were used as these are biologically relevant membranes that possess phospholipid asymmetry and are abundant in cholesterol. Upon rapid removal of cholesterol from the ghosts, the negative surface charge density of the inner surface showed a marked increase in the negative charge density as monitored with a fluorescently tagged polybasic peptide. Second, the internalization of PtdSer could be reduced by increasing the ionic strength of cytosol. In these experiments, the increase of cytosolic cations efficiently shield the negatively charged headgroups of the anionic phospholipids and thus limit the repulsive forces. Third, cells were subjected to low osmolarity conditions to produce a sustained mild stretching of the PM. This maneuver increased lateral tension and limited the internalization of PtdSer. Finally, the addition of exogenous PtdSer to cells also stimulated the internalization of PtdSer in the absence of any other signals. Taken together these results suggest that cholesterol depletion induces an increase in negative charge density, resulting in enhanced headgroup repulsion and spontaneous membrane curvature.

While the use of cyclodextrin is rather extreme, the experiments reveal essential biophysical properties of the PM. Specifically, charge repulsion by anionic headgroups is an important contributor to spontaneous curvature. It is worth noting that a variety of enzymes that can increase localized negative charge density are reported to positively regulate endocytosis such as lipid kinases, phospholipase D and PtdSer flippases [11-13]. Other recent publications have demonstrated that reducing membrane tension can promote membrane deformation and promote endocytosis [14, 15]. In the Zhi et al., study the authors demonstrated *in vitro* that a reduction in membrane tension in giant liposomes promoted membrane curvature [14]. They concluded that cellular membrane shapes and dynamics could be controlled by interacting with curvature-coupling proteins and through the regulation of membrane tension and lipid shape. The concept of altered membrane tension and fluidity was also examined in the context of increasing polyunsaturated fatty acyl chains. The study of Pinot et al. demonstrated that increasing docosahexaenoic acid (22:6 fatty acid) could overcome biophysical barriers and promote membrane tubulation and vesiculation mediated by endophilin and dynamin [15]. Finally, the delivery of membrane via exocytosis could provide “slack” and thereby decrease membrane tension. This concept would also help to explain the ultrafast endocytosis recently described to occur in the mouse hippocampal synapses [16]. Indeed, Watanabe and colleagues suggested that exocytosis leads to the concomitant internalization of roughly the same amount of membrane via ultrafast endocytosis. Overall, we suspect that nano- or microscale changes in biophysical properties may well influence a variety of naturally occurring vesicular transport process.

Our study demonstrated that increasing the negative charge density causes the electrostatic repulsion of the anionic headgroups which in turn promote spontaneous curvature. Similarly, several recent studies have highlighted the interplay between membrane tension and curvature. The generation of spontaneous curvature could be a significant contributor to many endocytic processes especially ultrafast endocytosis seen in the synapse.

**Figure 1.**
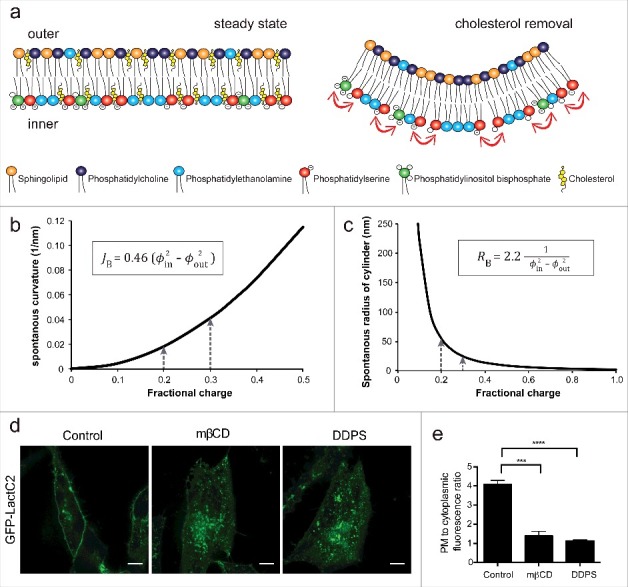
Increased charge density enhances spontaneous membrane curvature. a) A diagram depicting a model plasma membrane before (left) and after (right) cholesterol extraction. The color of the headgroup corresponds to each phospholipid, and the arrows indicate headgroup charge repulsion. b) Spontaneous bilayer curvature predicted theoretically. *J_B_*, a spontaneous curvature of a bilayer (nm); Φ in, the fraction of charged lipids in the inner monolayer and Φ out, the fraction of charged lipids in the outer monolayer. c) Calculated spontaneous preferred cylinder radius of a bilayer (*R_B_*) with varying fraction of negatively charged lipids in the inner monolayer. d) HeLa cells expressing the PtdSer probe, GFP-LactC2, were incubated with 10 mM mβCD or supplemented with 30 μM didecanoyl PtdSer (DDPS) 15 min and imaged using confocal microscopy. e) Quantitation of the ratio of PM to cytoplasmic GFP-LactC2 signal in control cells, mβCD- or DDPS-treated cells. Values represent means ± s.e.m., n  =  32 ***p < 0.005 and ****p < 0.001.

## References

[1] Panatala R, Hennrich H, Holthuis JC Inner workings and biological impact of phospholipid flippases. J Cell Sci. 2015;128:2021–32. doi:10.1242/jcs.102715 25918123

[2] Fairn GD, Grinstein S Cell biology. Precursor or charge supplier? Science. 2012;337:653–4. 22879491 10.1126/science.1227096

[3] Yang ST, Kreutzberger AJB, Lee J, et al. The role of cholesterol in membrane fusion. Chem Phys Lipids. 2016;199:136–43. doi:10.1016/j.chemphyslip.2016.05.003 27179407 PMC4972649

[4] Leventis PA, Grinstein S The distribution and function of phosphatidylserine in cellular membranes. Annu Rev Biophys. 2010;39:407–27. doi:10.1146/annurev.biophys.093008.131234 20192774

[5] Cho KJ, van der Hoeven D, Zhou Y, et al. Inhibition of Acid Sphingomyelinase Depletes Cellular Phosphatidylserine and Mislocalizes K-Ras from the Plasma Membrane. Mol Cell Biol. 2015;36:363–74. 26572827 10.1128/MCB.00719-15 PMC4719297

[6] Hirama T, Das R, Yang Y, et al. Phosphatidylserine dictates the assembly and dynamics of caveolae in the plasma membrane. J Biol Chem. 2017;292:14292–307. doi:10.1074/jbc.M117.791400 28698382 PMC5572903

[7] Fairn GD, Schieber NL, Ariotti N, et al. High-resolution mapping reveals topologically distinct cellular pools of phosphatidylserine. J Cell Biol. 2011;194:257–75. doi:10.1083/jcb.201012028 21788369 PMC3144401

[8] Maekawa M, Fairn GD Complementary probes reveal that phosphatidylserine is required for the proper transbilayer distribution of cholesterol. J Cell Sci. 2015;128:1422–33. doi:10.1242/jcs.164715 25663704

[9] van Meer G, Voelker DR, Feigenson GW Membrane lipids: where they are and how they behave. Nat Rev Mol Cell Biol. 2008;9:112–24. doi:10.1038/nrm2330 18216768 PMC2642958

[10] Hirama T, Lu SM, Kay JG, et al. Membrane curvature induced by proximity of anionic phospholipids can initiate endocytosis. Nat Commun. 2017;8:1393. doi:10.1038/s41467-017-01554-9 29123120 PMC5680216

[11] Shen H, Giordano F, Wu Y, et al. Coupling between endocytosis and sphingosine kinase 1 recruitment. Nat Cell Biol. 2014;16:652–62. doi:10.1038/ncb2987 24929359 PMC4230894

[12] Rankovic M, Jacob L, Rankovic V, et al. ADP-ribosylation factor 6 regulates mu-opioid receptor trafficking and signaling via activation of phospholipase D2. Cell Signal. 2009;21:1784–93. doi:10.1016/j.cellsig.2009.07.014 19666113

[13] Pomorski T, Lombardi R, Riezman H, et al. Drs2p-related P-type ATPases Dnf1p and Dnf2p are required for phospholipid translocation across the yeast plasma membrane and serve a role in endocytosis. Mol Biol Cell. 2003;14:1240–54. doi:10.1091/mbc.E02-08-0501 12631737 PMC151593

[14] Shi Z, Baumgart T Membrane tension and peripheral protein density mediate membrane shape transitions. Nat Commun. 2015;6:5974. doi:10.1038/ncomms6974 25569184 PMC4353700

[15] Pinot M, Vanni S, Pagnotta S, et al. Lipid cell biology. Polyunsaturated phospholipids facilitate membrane deformation and fission by endocytic proteins. Science. 2014;345:693–7. 25104391 10.1126/science.1255288

[16] Watanabe S, Rost BR, Camacho-Perez M, et al. Ultrafast endocytosis at mouse hippocampal synapses. Nature. 2013;504:242–7. doi:10.1038/nature12809 24305055 PMC3957339

